# Normal salivary production using a swab method in clinical settings

**DOI:** 10.1111/coa.13953

**Published:** 2022-07-12

**Authors:** Patrick Rønde Møller, Mathias Lyngbye Kærsgaard, Jolanta Grydehøj, Therese Ovesen

**Affiliations:** ^1^ Department of Otorhinolaryngology Aalborg University Hospital Aalborg Denmark; ^2^ Clinic for Rheumatology, Department of Internal Medicine Regional Hospital West Jutland Holstebro Denmark; ^3^ University Clinic for Flavour, Balance and Sleep Department of Otorhinolaryngology, Regional Hospital West Jutland Holstebro Denmark; ^4^ Department of Clinical Medicine Aarhus University Aarhus Denmark

**Keywords:** dry mouth, hyposalivation, salivary flow rate, salivary gland disease, salivary gland dysfunction, xerostomia

## Abstract

**Objectives:**

The purpose of the study was to generate age‐ and gender‐based normative data for unstimulated salivary flow rate (uSFR) by means of a swab method, and to provide preliminary results of using the test in patients suspected of reduced salivary function.

**Methods:**

The 130 healthy participants without subjective xerostomia or suspicion of reduced salivation were recruited. Measurements of uSFR were conducted three times per subject and mean uSFR was calculated for the entire population and stratified according to age and gender. The method was applied in a pilot population of 25 patients suffering from either Sjögren's syndrome or had underwent irradiation of the head and neck.

**Results:**

Mean uSFR in the healthy group was 0.808 g/min (range: 0.165–2.442). Not significant trends towards declining uSFR with increasing age and higher uSFR in women were seen. Mean uSFR in the patients was 0.429 g/min (range: 0.111–1.448), which was significantly lower than normative values. Use of xerogenic drugs correlated to lower uSFR.

**Conclusion:**

Age‐ and gender‐based normative data of uSFR was presented using a fast and readily implementable swab test. The test was able to objectively verify hyposalivation among patients suffering from Sjögren's syndrome or having been exposed to head and neck radiation.

**Key Points**

- The swab method is a fast and reliable method for measuring unstimulated salivary flow rate (uSFR).
- The swab method is able to distinguish between healthy subjects and diseased patients.
- There is no significant difference in uSFR between healthy male and female subjects.
- Use of medication may greatly increase risk of hyposalivation.
- Further research into measurement of unstimulated and stimulated salivary flow rate is needed.

## 1 INTRODUCTION

The terms, xerostomia and hyposalivation, are often used in relation to each other and sometimes interchangeably,^1^ though they are not the same. On the other hand, they may occur simultaneously. Xerostomia describes the sensation of dry mouth and is a frequent complaint in the elderly population.^2^ Hence, xerostomia is considered a condition related to old age, but also younger adults may present with xerostomia.^3^, ^4^ Hyposalivation or salivary gland dysfunction (SGD) describes reduced production of saliva.^1^ The reason of SGD may be old age, but other underlying causes include medication, rheumatologic disease, such as Sjögren's syndrome, radiotherapy of the head and neck, and salivary gland diseases.^5^, ^6^, ^7^ Clear distinction between xerostomia and SGD may be important in terms of treatment. Xerostomia without SGD is treated with non‐invasive agents, that is, saliva replacement fluid, whereas SGD may be subject to more invasive modalities such as electrostimulation of the salivary glands.^8^ Transplantation of mesenchymal stem cells^9^ is also under investigation to improve salivary gland function in patients with SGD.^10^ Hence, cases of SGD may require further diagnostic measures to explore possible treatments.

Currently, xerostomia is either merely registered as a subjective complaint or by means of the Xerostomia Inventory List.^2^ Sialometry is a more objective method and may be performed when SGD is suspected and can differentiate between xerostomia and SGD.^11^ However, several problems relate to sialometry as various and often time consuming methods are used,^12^ and normative data is lacking. Interpretation of sialometric results should also take age and gender into account as it is known that unstimulated salivary flow rate (uSFR) decreases with age and may differ between genders.^13^

In summary, there is a strong need for a simple, readily interpretable method for quantification of salivation providing age‐ and gender‐adjusted normative data in order to differentiate pathological uSFR from normal values and thereby allocate candidates for relevant treatment.

In the present study, we introduce a standardised, fast, and easy sialometric method for daily clinical settings. Age‐ and gender‐adjusted normative data is presented along with measures among patients suffering from Sjøgren's syndrome as well as patients treated with radiotherapy due to head and neck cancer.

## 2 SUBJECTS AND METHODS

### 2.1 Subjects

Subjects with presumably healthy salivary glands (non‐SGD) were recruited from staff and patients from two different departments of otorhinolaryngology. Subjects were instructed to avoid eating and drinking for 2 h prior to measurements, including use of chewing gum and smoking. Exclusion criteria included previous radiotherapy of the head and/or neck, chemotherapy, autoimmune diseases, salivary gland diseases, psychiatric/neurological diseases/medication and/or cognitive impairment. Age, gender and medical use was registered. To obtain information about the reliability of the swab method, 10 normal subjects were tested twice with 2 days interval.

Patients with SGD were recruited partly from the outpatient clinic at a department of rheumatological diseases and partly from a department of oncology. Patients with Sjögren's syndrome were prioritised as well as patients treated with full dosage head and neck radiation.

Measurements of uSFR were made using a modification of the swab method suggested by Navazesh.^12^ The subject was instructed to cleanse the oral cavity with demineralized water. After cleansing, a pre‐weighed Abena Curi‐Med non‐woven swab (7.5 × 7.5 cm) was folded and placed under the subject's tongue for 1 min. The subject was instructed to close the mouth and not talking or swallowing. The swab was immediately weighed after 60 s and subsequently discarded. The measurement was repeated for a total of three times for each subject. The weight was noted in gram (g) with three decimals using Kern Precision Balance PFB‐300‐3. Thus, the calculated mean uSFR was g/min.

#### 2.1.1 Statistics

Statistical analyses were conducted using STATA for Windows version 16.0 (STATA CORP LD, College Station, TX). A conventional p‐value of 0.05 was used as the criterion for statistical significance.

All non‐SGD subjects were divided into groups based on age, with a ten‐year interval of all groups. An exception was made to the youngest group that contained subjects with ages between 18 and 29 years. For each subject, the mean uSFR based on the three measurements was calculated. The use of xerogenic drugs was noted, and the percentage of subjects using one or more xerogenic drugs was calculated (XD). Simple descriptive statistics are presented including 95% CI for uSFR (g/60 s) for all non‐SGD age groups and both genders. All data was analysed for normal distribution by Shapiro‐Wilks Test.The Wilcoxon Rank Sum test (WRS) was applied to test age and gender differences between the healthy population and the patients as well as to compare test–retest results.

## 3 RESULTS

### 3.1 Normative values of uSFR

In total, 130 non‐SGD subjects were recruited (69 females and 61 males) with a mean age and SD of 48.14 ± 16.83 years. Measurements of uSFR and descriptive statistics for each age and gender group of non‐SGD subjects are shown in Table 1, along with 95% confidence intervals. Xerogenic drugs (e.g beta blockers, selective serotonin reuptake inhibitors [SSRIs], and analgesics such as morphine and acetaminophene)^14^ were used on a daily basis by a total of 34%.

**TABLE 1:** Unstimulated salivary flow rate in healthy subjects

Age group	Gender	*N*	XD% (*N*)	Mean	SEM	Min	Max	95% CI	
18–29 years	F	10	30 (3)	1.005	.256	.165	2.442	.427	1.583
	M	10	30 (3)	.791	.096	.312	1.330	.574	1.008
30–39 years	F	14	7.1 (1)	.902	.160	.166	2.390	.557	1.247
	M	10	10 (1)	.694	.100	.365	1.389	.468	.920
40–49 years	F	14	28.6 (4)	.831	.152	.205	1.993	.501	1.160
	M	10	40 (4)	.822	.087	.471	1.445	.626	1.019
50–59 years	F	10	20 (2)	.716	.104	.234	1.272	.481	.951
	M	10	40 (4)	.831	.092	.407	1.154	.624	1.038
60–69 years	F	11	45.4 (5)	.815	.119	.420	1.780	.550	1.080
	M	10	40 (4)	.691	.080	.337	1.133	.509	.872
70–79 years	F	10	60 (4)	.821	.147	.387	1.654	.588	1.153
	M	11	72.7 (8)	.736	.124	.198	1.552	.459	1.014

Abbreviations: CI, confidence intervals; SEM, standard error of the mean; XD, percentage using xerogenic drugs.

**FIGURE 1:**
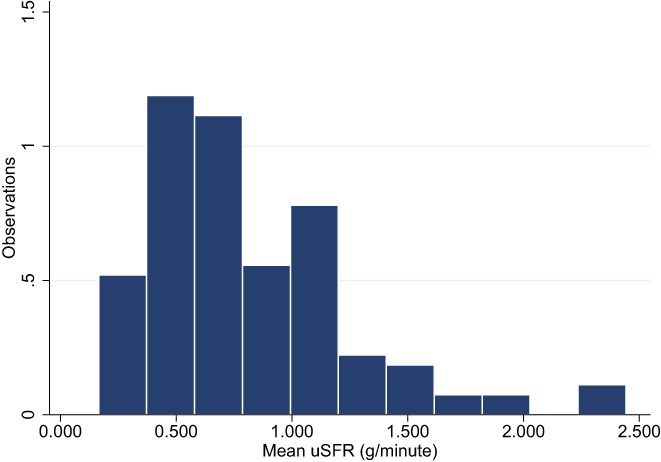
Distribution of mean unstimulated salivary flow rate (uSFR) (g/min).

The overall mean of uSFR for non‐SGD subjects was 0.808 g/min (SEM: 0.039, 95% CI: 0.731, 0.885). The median was 0.771 g/min. The data set is depicted in Figure 1, showing that it is left skewed confirmed by Shapiro‐Wilks Test (z = 5.231, *p* > 0.0001).

### 3.2 Difference in non‐drug users and users of xerostomic drugs

Of the healthy subjects, a total of 45 (34%) used one or more potentially xerostomic drugs (XD), as shown in Table 2. Using WRS to compare users of XD to non‐users, we found no statistically significant difference between the two groups (*z* = 1.725, *p* = 0.085).

**TABLE 2:** Difference in unstimulated salivary flow rate (uSFR) between healthy subjects with or without use of xerogenic drugs

XD	*N*	Mean age (years)	Mean uSFR (g/min)	SEM	95% CI	
Yes	85	45.1	0.859	0.473	0.757	0.961
No	45	56.8	0.711	0.375	0.599	0.824

Abbreviations: CI, confidence intervals; SEM, standard error of the mean; XD, use of xerogenic drugs.

**FIGURE 2:**
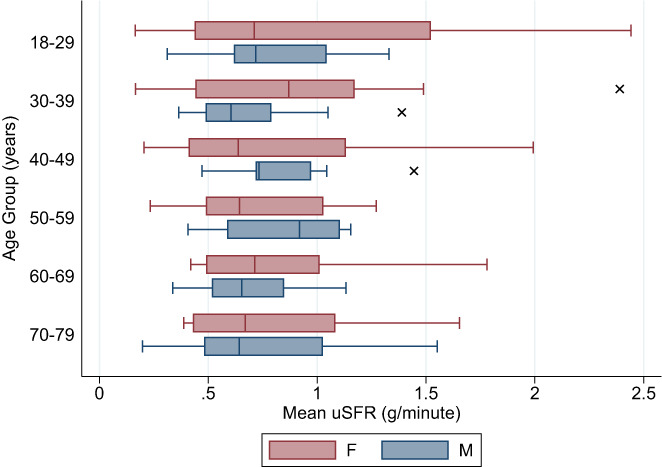
Box plot showing mean ranges of unstimulated salivary flow rate (uSFR) (g/min).

**FIGURE 3:**
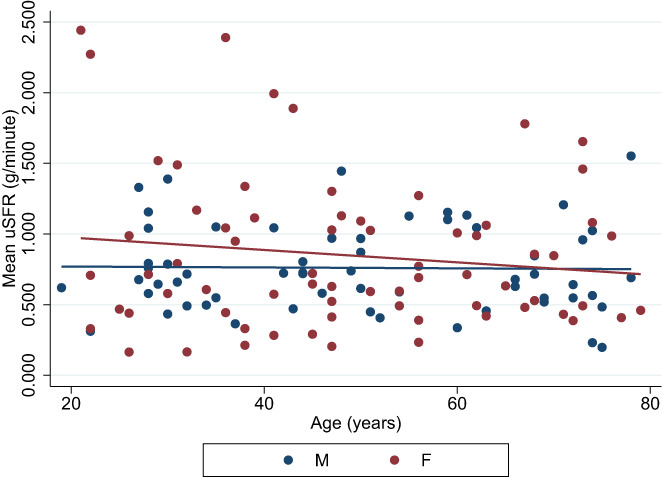
Linear regression of mean values of unstimulated salivary flow rate (uSFR) (g/min) according to gender.

The mean scores for each age group were between 0.691 and 1.005 g/min with little variance between genders (Figure 2). The overall female mean was 0.850 g/min (SEM: 0.065, 95%CI: 0.720, 0.979), and the overall male mean was 0.760 g/min (SEM: 0.039, 95%CI: 0.682, 0.839). No statistically significant difference was found between overall female and male means (*z* = −0.049, *p* = 0.961). Female mean values were higher in younger individuals compared to males of same age group but declined faster with age (Figure 3).

In general, uSFR declined with increasing age. Using the age group between 18 and 29 years as baseline, WRS was performed, comparing each group to baseline. No statistically significant difference was found between either group compared to baseline (30–39 years: *p* = 0.786, 40–49 years: *p* = 0.925, 50–59 years: *p* = 0.808, 60–69 years: *p* = 0.715, 70–79 years: *p* = 0.566).

### 3.3 Difference between SGD and non‐SGD groups

Of the 25 patients with SGD, 19 were females and six were males. The female patients suffered from Sjøgren's syndrome, while the six male patients had received radiotherapy of the head and/or neck. The overall mean uSFR of the SGD patients was 0.429 g/min (SEM: 0.055, min: 0.111, max: 1.448, 95% CI: 0.314, 0.544). Comparison between uSFR values in the non‐SGD and SGD‐groups, showed statistically significant lower uSFR in the patients (*z* = 4.695, *p* < 0.0001).

**FIGURE 4:**
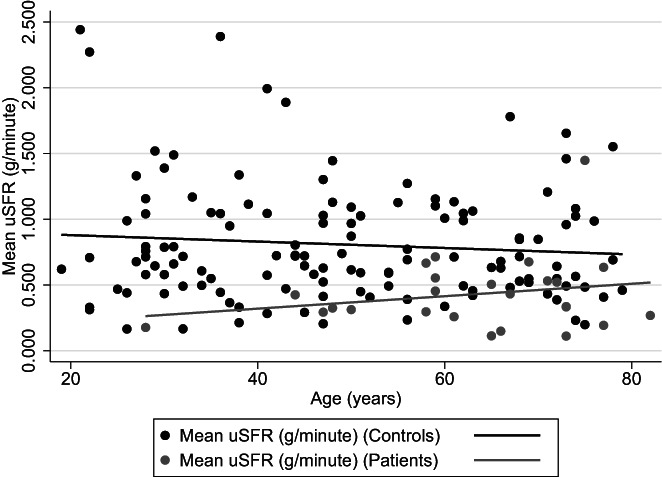
Linear regression of mean values of unstimulated salivary flow rate (uSFR) (g/min) in patients compared to healthy controls.

The SGD group showed an increase in uSFR with increasing age approaching the levels in the non‐SGD group (Figure 4).

### 3.4 Test–retest of the swab method

Of the 10 healthy subjects, five were male and five were female. Ages differed from 23 to 75 years. The overall mean of the first sample was 1.175 g/min (SEM: 0.183, min: 0.434, max: 2.184, 95% CI: 0.762, 1.589). The overall mean of the second sample was 1.133 (SEM: 0.167, min: 0.503, max: 2.033, 95% CI: 0.756, 1.509). The samples did not show statistically significant differences (*z* = 1.274, *p* = 0.203).

## 4 DISCUSSION

This is the first study to define normative, age‐and gender‐adjusted measurements of uSFR in non‐SGD subjects using a modified swab method. The method demonstrated good test–retest reliability and the test was able to differentiate patients from healthy controls; hence, it can be used as baseline for easy and fast quantification of uSFR in clinical settings.

We found a non‐significant trend towards declining uSFR's with age, and that use of medication with the potential of inducing hyposalivation and xerostomia was more prevalent among the older age groups compared to the younger. Thus, the reduction of uSFR with increasing age may partly be explained by medication, which is consistent with several other studies.^13^, ^14^, ^15^ On the other hand, the group of subjects using potentially xerogenic medication had no subjective complaints of xerostomia and comparing non‐drug users to users of one or more XD showed no significant difference between the two groups. When comparing this study to similar studies measuring uSFR, we find that our results are consistent. Age has not been shown directly to be a risk factor of hyposalivation, but studies suggest that age‐related conditions may alter uSFR.^16^ That is, age‐related chronic conditions may increase the amount of pharmacological therapy, and thereby contribute to hyposalivation. Coherent with our study, other studies have shown no significant difference in uSFR in younger individuals (<50 years), and that female gender, age over 65 years and drug intake significantly increase risk of hyposalivation.^16^, ^17^, ^18^

Our study found non‐significant differences between male and female uSFR. uSFR in younger females were higher than uSFR in younger males, but female uSFR declined more rapidly with age than male uSFR. Several studies^19^, ^20^, ^21^, ^22^ have shown that menopause and hormonal changes are risk factors in developing xerostomia and hyposalivation, which may explain the difference in decline of uSFR between the two genders in this study. Other studies have shown no significant difference between male and female uSFR as well, though one study has shown higher male uSFR compared to female.^16^ We found no studies investigating the role of testosterone on salivary production.

Our study focused on uSFR, but unlike many other studies, we used the swab method. Flink et al.^16^ used the spitting method proposed by Navazesh,^12^ and found generally lower uSFR values compared to those obtained in our study (n: 1420, mean: 0.29 ml/min SD: 0.24). Under the assumption that 1 ml of saliva equals 1 g of saliva, our mean uSFR is almost triple of the mean reported by Flink et al. The reason for this discrepancy may be that the swab placed under the tongue causes mechanical manipulation of the submandibular gland, which in turn leads to increased uSFR.^12^

Other types of measurement of salivary gland function include stimulated salivary flow rate (sSFR), in which the subject is instructed to either chew a tasteless substance or is stimulated with agents such as citric acid. None of these sSFR studies has used the swab technique. Instead, several studies have used a modified version of the suction method^12^ by using custom made devices placed over the Wharton duct in order to collect saliva.^23^, ^24^, ^25^ However, such methods are time consuming and rather unpleasant for the patients, which makes them incompatible with daily clinical settings. This study opted out of investigating the swab method to measure sSFR, but we see no restrictions for the usage. Woods et al.^26^ used chewable cotton swabs to collect saliva samples for analysis, a method that would be comparable to the swab method if small changes were made.

The swab method is not suitable for exact measures of saliva production but as the technique is fast and readily performed, it may be used as a screening tool in outpatient clinics. Under the same conditions, the results of a re‐test were not significantly different from the primary test. This means, that the swab test can be used to follow individuals over time as well as comparing results before and after various interventions. Patients with abnormal uSFR values by the swab method could undergo more exact and time‐consuming techniques for comparison, such as the spitting method suggested by Navazesh.^12^ Importantly, the swab method was able to differentiate patients and healthy controls.

A large part of the non‐SGD subjects was recruited from staff of the Departments of Otorhinolaryngology at the Aalborg University Hospital and the Regional Hospital West Jutland. One could argue that it carries a significant risk of selection bias, especially since the health status of hospital staff could be expected to be higher than the background population. However, studies suggest that health care workers and their patients show the same prevalence and outcome in chronic and infectious diseases.^27^

The study reflects findings in uSFR from 130 non‐SGD subjects, measured a total of three times. It is likely that investigations under similar conditions will provide similar results, although the external validity needs to be tested in future studies. In addition, the internal validity could be explored by comparing the swab method with other available methods.

For diagnostical purposes, the study provides an excellent tool for the clinician to determine whether a patient shows signs of hyposalivation, or if the complaints are more likely to stem from xerostomia. This could lead to earlier and more precise diagnostics of several conditions, making earlier intervention possible. For research purposes, our study contributes with a standardised and comparable method to investigate unstimulated saliva production in general, as well as studies of preventing conditions related to hyposalivation such as caries, sialolithiasis, and reduced sense of taste.

## 5 CONCLUSION

This study has provided a tool for fast and easy measurements of uSFRs in clinical settings, and it has provided a series of age‐ and gender‐based normative data. Thus, the method is easily implemented as routine testing in outpatient clinics.

## AUTHOR CONTRIBUTIONS

Patrick Rønde Møller conceived and designed the study, collected data, analysed data, and wrote the paper. Mathias Lyngbye Kærsgaard collected data for healthy subjects. Jolanta Grydehøj collected data and assisted in recruitment of patients with salivary gland disease. Therese Ovesen conceived and designed the study and gave contributions in writing the paper.

## FUNDING INFORMATION

This research received no specific grant from any funding agency in the public, commercial, or not‐for‐profit sectors.

## CONFLICT OF INTEREST

No conflicts of interest were found in this study.

## ETHICS STATEMENT

The study was approved by the The North Denmark Region Committee on Health Research Ethics.

## Data Availability

Data is available upon request from the corresponding author.
